# Numerical simulation of nanopost-guided self-organization dendritic architectures using phase-field model

**DOI:** 10.1371/journal.pone.0199620

**Published:** 2018-07-02

**Authors:** You-Ren Hsu, Ming-Chieh Lin, Hua-Kai Lin, Yu-Hsu Chang, Chih-Cheng Lu, Hua-Yi Hsu

**Affiliations:** 1 Institute of Nanoengineering and Microsystems, National Tsing Hua University, Hsinchu, 30013, Taiwan; 2 Department of Electrical and Biomedical Engineering, Hanyang University, Seoul, 04763, Korea; 3 Department of Chemical Engineering, National Taiwan University, Taipei, 10617, Taiwan; 4 Department of Materials and Mineral Resources Engineering, National Taipei University of Technology, Taipei, 10608, Taiwan; 5 Department of Mechanical Engineering, National Taipei University of Technology, Taipei, 10608, Taiwan; Queen’s University at Kingston, CANADA

## Abstract

Self-organized dendritic architecture is of fundamental importance and its application can be used in many natural and industrial processes. Nanopost arrays are usually used in the applications of reflecting grating and changing the material surface wettability. However, in recent research, it is found that nanopost arrays can be fabricated as passive components to induce the dendritic self-organizaed hierarchical architectures. Via this simplified Phase-Field based finite element simulation, the surface dendritic self-organized architecture morphology and expanding speed in the growing path can be controlled by nanopost structures. In addition, nanopost array arrangement on the surface affects the hierarchal architecture branching distribution. Finally, with an external applied force introduced to the system, it enables the nanopost as an active component. It is found that nanopost surroundings significantly impact the final distribution of dendritic architectures which is qualitatively in agreement with experiments and induce these dendritic architectures to form assigned character patterns after the external driving forces are introduced into the system. This novel study can fundamentally study the dynamic physics of dendritic self-organized architecutes provide an indicator for the development of smart self-organized architecture, and a great opportunity for the creation of large-scale hierarchical structures.

## Introduction

The formation and control of self-organized dendritic structure on microstructure has attracted a lot of attention in recent years [1–8]. The interest is due to the low cost, simple operation, and the spontaneous self-organization of the formation process. Self-assembly technology can assemble molecules of a special nature from a disordered state into an ordered construction without any external driving forces. It has been massively applied as sensing structures in manufacturing of electronic components and sensors. However, the fabrication of self-organized structure usually comes with various morphologies under different conditions. Whether the structures can be formed through appropriate induction remains to be determined. Also, the branching direction and angles among these branches are usually arbitrary and lack control due to the nature of random aggregation, which makes pattern predicition difficult and limits the application of such structures.

Among self-organized architectures, the dendritic structures are commonly found and observed in various structure formations [9–14]: such as organism blood vessels, leaf venation, organic aggregation, alloy melting structures, etc. Dendritic structure can be found in biomimetic structures with large surface areas, and it has been applied to improve the sensibility of biosensors [15]. In addition, dendritic structure, as a biomimetic structure, has great potential to be applied into tissue culture. Dendritic structure, like self-organization, usually comes with a phase change and anisotropic structure formation. However, it has been a challenge to develop a simple synthetic approach to create external morphology-controlled hierarchical architectures of various systems. Previous research has attempted to form and observe a structure by using an extremely accurate instrument and measuring the material properties on a tiny scale. However, some limitations still exist for observing these structures. The limitations of scanning electron microscopy and transmission electron microscopy are that a vacuum environment is required and that structure observation is generally conducted under a static condition [2]. Thus, a dynamic condition cannot be observed. Only a few indirect experiments can be performed to speculate how these structures are formed. Therefore, numerical analysis study offers an opportunity and efficient guidance in the phase change problem.

The self-assembly of a structure typically occurs in nanoscale. However, through a nanopost structures guided mechanism, the self-organized architecture was observable on a microscale [2], which is relatively large. This is unique among previous research about self-organized architectures. Experimentation has revealed the distribution of the branching angle of microscale dendritic structure can be induced by arrangement of nanopost arrays. Through this multi-scale problem, different approaches should be considered. In addition to these experimental studies, the computing simulations and numerical methods have become efficient techniques in science which can provide much more information during the microstructural evolution as well as phase and temperature distributions during the self-organization process. Molecular dynamics (MD) is capable of solving the nanoscale problems such as protein folding [16] and grain boundaries faceting under atomic resolution [17]. However, it is time and computationally expensive. The phase-field (PF) method is able to capture the mesoscale physics based on free energy of the system and average short time and length scales. The phase-field-crystal (PFC) model is valid in between the MD and PF method, and the free energy does not average over atomic distance, resulting in pattern formation at equilibrium [18].

Phase field modeling approach has been commonly used for microstructure formation in material science and polymer studies [19–21]. It is also efficient in solving this multi-scale nanopost-guided self-organized dendritic architecture study. In 1992, Kobayshi was the first one to propose the simple phase field model and study dendritic crystal growth which includes the anisotropy [22]. Karma used phase field model to study time-dependent free-boundary problem of single dendritic crystallization of a pure melt using thin-interface limit [23]. Both of the two studies are based on the finite difference method (FDM). Recently, a modified phase field model had been used for studying and understanding snow crystal growth in three dimensions [24]. As to polymer science, Ross used self-consistent field theory simulation to explore the graphoepitaxy of spherical morphology block copolymers templated by an array of posts [19]. Li reported the self-assembly of block copolymer–homopolymer blends in bulk, as well as under the direction of periodic patterned surfaces by computer simulations of the time-dependent Ginzburg–Landau theory [20]. Zhang uses large cell simulations of self-consistent field theory to study self-assembly behaviors of cylinder-forming diblock copolymers directed by an array of anisotropic nanoposts with elliptical shape, which can be an indicator of fabricating well-ordered nanostructures of block copolymer lithography [21].

The methods involved in microstructure formation can be categorized into two types: (1) active: using external force to change the morphology of molecules or material [25], such as using electric field to control the arrangement of molecules, or liquid alloy under super cool condition [26]; (2) passive: material structure being formed through pre-tailored configurations [2]. The main goal of this study is to develope a generalized framework for studying dendritic architectures controlled by tailored passive geometric structures and/or active drives. Therefore, in this work, the Karma’s phase field model coupled with the energy equation has been extended to include external excitations and arbitrary geometric confinement using finite element method (FEM), which is considered more accurate than the FDM. For the first time, this integrated model can be used to investigate the microscale evolutions under nanoscale constraints, i.e., a multi-scale problem, on adaptive meshes. Additionally, an external excitation such as electrical field, magnetic field, and thermal source/sink can be applied to the system to study its controllability of structure formation. A self-assembly study can be extensively applied to various nanomaterials to determine the influence of the self-organized process, such as the addition of dielectric particles in the coating solution to form a solid film that can facilitate energy saving. For demonstration, through the extended phase field model, the temperature field is assumed to be the only dominant excitation as the active source to promote the dendritic structure formation, and circular/elliptical nanopost arrays are employed as the passive confinement for guiding the configuration. The simulation result is qualitatively in agreentment with the experimental results. It is demonstrated that this framework is general and serves as a foundation for realizing controllable large-scale pattern formation at lower cost and energy consuming.

## Model and theory

The phase-field approach is rapidly emerging as a method of choice for simulating interfacial pattern formation in solidification and phase change problem. The thin-interface self-organized dendritic growth model used here was proposed by Karma and Rappel [23]. To describe the growth of dendritic structure, *ψ* is defined as the phase state of the structure. The isotropic growth is from liquid state *ψ* = −1 to solid state *ψ* = 1, and the interface of the solid and liquid is *ψ* = 0. The governing equations of the extended phase field model coupled with energy equation are written as:

$$\begin{array}{c} \tau \left(n\right)\frac{\partial \psi }{\partial t}=W{\left(n\right)}^{2}\nabla^{2}\psi -\frac{\partial F(\psi ,\lambda u)}{\partial \psi }, \end{array}$$ (1)

$$\begin{array}{c} \frac{\partial u}{\partial t}=D\nabla^{2}u+\frac{\partial h(\psi )/2}{\partial t}+S, \end{array}$$ (2)

where $n=\vec{\nabla }\psi /\left|\vec{\nabla }\psi \right|$, *ψ* is the state of phase which varies from *ψ* = −1 in the liquid to *ψ* = 1 in the solid across an interface region of thickness *W*, and *τ* is a characteristic time of attachment of atoms at the interface. Here, *W* and *τ* are assumed anisotropic. Because the liquid and solid states are stable states in this system, the *F*(*ψ*, λ*u*) must have a minimum value at *ψ* = 1 or *ψ* = −1. It is assumed that *F*(*ψ*, λ*u*) ≡ *f*(*ψ*) + λ*g*(*ψ*)*u* is a mathematical function with the form of a double-well potential, where the relative height of two minima is temperature dependent, λ is a dimensionless parameter that controls the strength of the coupling between the phase and the diffusion fields, *u* = (*T* − *T*_*M*_)/(*L*/*C*_*p*_) represents the dimensionless temperature field, *T*_*M*_ is the melting temperature, *L* is the latent heat of melting, *c*_*p*_ is the specific heat at constant pressure, *D* is the thermal diffusivity, *h*(*ψ*) is a function describes the generation of latent heat, and specially *S* is added to extend the Karma’s PF model as an external energy source excitation of choice. The double-well potential *f*(*ψ*) is usually taken as this simple form [23],

$$\begin{array}{c} f\left(\psi \right)=-\frac{\psi^{2}}{2}+\frac{\psi^{4}}{4}, \end{array}$$ (3)

and for computational purpose,

$$\begin{array}{c} g\left(\psi \right)=\psi -\frac{2\psi^{3}}{3}+\frac{\psi^{5}}{5}, \end{array}$$ (4)

The generation of latent heat function *h*(*ψ*) is taken as

$$\begin{array}{c} h(\psi )\equiv \psi . \end{array}$$ (5)

From here, the phase field model for dendritic growth is described in 2D. A standard four-fold anisotropy is defined by

$$\begin{array}{c} W\left(n\right)=W_{0}a_{s}\left(n\right) \end{array}$$ (6)

where the underlying cubic symmetry of the surface energy is expressed by *a*_*s*_(**n**),

$$\begin{array}{c} a_{s}\left(n\right)=\bar{a_{s}}\left[1+\epsilon^{\prime }\left(n_{x}^{4}+n_{y}^{4}\right)\right]=\bar{a_{s}}\left[1+\epsilon^{\prime }\left(\cos^{4}\bar{\theta }+\sin^{4}\bar{\theta }\right)\right], \end{array}$$ (7)

Here $\bar{\theta }$ is the angle between the normal to the interface and some fixed crystalline axis. In this work, *ϵ*_4_ is taken as the measure of the anisotropy. The relation between *ϵ*_4_ and $\bar{a_{s}}$ is defined by

$$\begin{array}{c} \bar{a_{s}}=\left(1-3\epsilon_{4}\right), \end{array}$$ (8)

$$\begin{array}{c} \epsilon^{\prime }=\frac{4\epsilon_{4}}{1-3\epsilon_{4}}, \end{array}$$ (9)

$$\begin{array}{c} W\left(n\right)=W_{0}\left(1-3\epsilon_{4}\right)\left[1+\frac{4\epsilon_{4}}{1-3\epsilon_{4}}\frac{{\left(\partial_{x}\psi \right)}^{4}+{\left(\partial_{y}\psi \right)}^{4}}{|\vec{\nabla }{\psi |}^{4}}\right], \end{array}$$ (10)

Hence the final forms of governing equations are:

$$\begin{array}{c} \frac{\partial u}{\partial t}=D\nabla^{2}u+\frac{1}{2}\frac{\partial h(\psi )}{\partial t}+S_{T}, \end{array}$$ (11)

$$\begin{array}{ll} \tau \left(n\right)\partial_{t}\psi & =\left[\psi -\lambda u\left(1-\psi^{2}\right)\right]\left(1-\psi^{2}\right)+\vec{\nabla }\cdot \left[W{\left(n\right)}^{2}\vec{\nabla }\psi \right]\\ & +\partial_{x}\left(|\vec{\nabla }\psi {|}^{2}W\left(n\right)\frac{\partial W\left(n\right)}{\partial \left(\partial_{x}\psi \right)}\right)\\ & +\partial_{y}(|\vec{\nabla }\psi {|}^{2}W\left(n\right)\frac{\partial W\left(n\right)}{\partial \left(\partial_{y}\psi \right)}), \end{array}$$ (12)

where *S*_*T*_ represents an external thermal excitation. The governing Eqs (11) and (12) are self-written and solved in a non-dimensional form using the adaptive finite element method as implemented in COMSOL Multiphysics. Based on this extended phase-field model, a set of coupled partial differential equations (PDE) has been constructed and solved for self-consistent solutions. In our previous research [27], a three dimensional simulation of dendritic self-organized structure growth had been carried out but it was extremely time consuming. In a real system of self-organized dendritic architecture, a thin film is usually formed between perpendicular nanoposts on a substrate [2]. Thus, the system can be simplified to a two-dimensional system and the interaction between the liquid and nanopost solid walls is neglected for simplification, assuming a non-wetting boundary condition. In addition, the formation of ordered patterns in microscale system with nanoscale post is considered in this study and the simulation domain must be much larger than the size of a single nanopost. Therefore, it becomes a extremely large scale problem to be solved in three dimensional. For demonstation of two dimensional modeling, the computational domain is a circular domain with radius r = 80 units. According to Karma’s [23], $\lambda =\tau_{0}D/a_{2}W_{0}^{2}$, and the simulations reported here are for the fixed values *W*_0_ = 0.25, *τ*_0_ = 1, *ϵ*_4_ = 0.05, *a*_2_ = 0.6267, and *D* = 4. More details can be found in Karma’s [23]. There are approximately 360,000 arbitrary triangular meshes generated in the simulation domain, and the mesh parameters are listed in Table 1. The adaptive meshes are generated according to manually defined regions, and so as the mesh sizes have been refined around the obstacles boundaries to prevent discretization errors within an affordable computational resource. The time step size Δ*t* = 0.01 is employed in all of the simulations. A zero-flux boundary condition is set at the outside circle boundary of the computational domain, and therefore, there is no energy exchange with the outside system. A small circular seed in a supercooled condition is used as the initial point. A step function is given for the initial phase profile so the phase-field variable *ψ* varies smoothly between 1 in the solid and -1 in the liquid, and the central region is assigned to be a seed in solid state(*ψ* = 1).

**Table 1:** Parameters of the mesh.

	unit
Maximum element size	0.18
Minimum element size	6.75E-06
Maximum element growth rate	1.2
Curvature factor	0.25
Resolution of narrow regions	1
Total mesh numbers	360000

## Results and discussion

Generally, it is accepted that fractal formation aries in non-equillibirum situations [28]. Dendritic architecture forms when there is driving force for the crystallation, such as high supercooling or high supersaturation [11, 29]. It has been shown that the thin film of liquid is important for branch structure formation [30, 31]. The migration of the liquid film propagates along the fractal structure tip, providing the driving force for transporting liquid to form a crystallized solid architecture.

**Fig 1:**
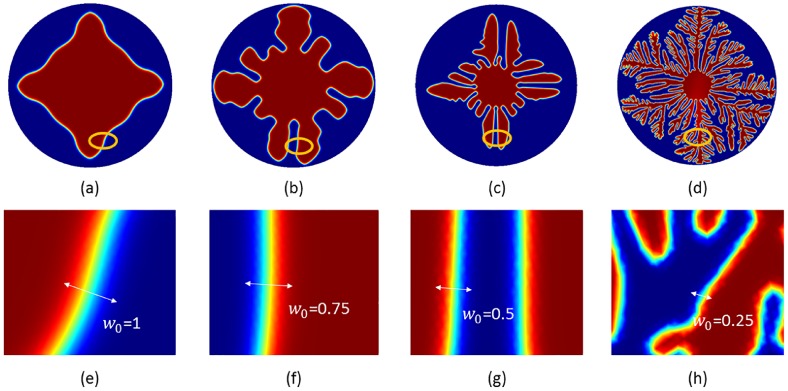
Dendritic structure at different interface thickness. (a)-(d) represent the dendritic structure self-organization growth at the same simulation conditions and the same growth time for *W*_0_ = 1, *W*_0_ = 0.75, *W*_0_ = 0.5, and *W*_0_ = 0.25, respectively. (e)-(h) are the enlarged views of (a)-(d), respectively, and it is seen that branching ability is getting weaker while the interface thickness *W*_0_ increases.

**Fig 2:**
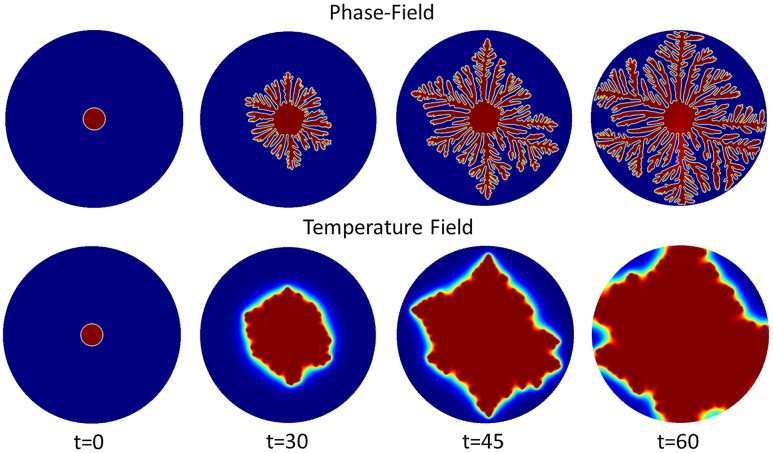
Phase field and corresponding temperature field of the dendritic structure at interface thickness *W*_0_ = 0.25 on the surface without nanoposts guided. At t = 0, the center region of the simulation domain is assigned to be solid state (*ψ* = 1). Due to the temperature difference of solid region and surrounding environment, there is a driving force which gives rise to dendritic architecture self-organization. As long as the temperature achieves the balanced temperature, the dendritic self-organization growth stops.

From the simulation results, it can been seen that the anisotropy *W*(**n**) = *W*_0_ *a*_*s*_(**n**) and *W*_0_ decides the interface thickness. From Fig 1(a)–1(d), *a*_*s*_(**n**) decides the branching direction of dendritic structure because 1 − *ϵ*_4_ ≤ *a*_*s*_(**n**) ≤ 1 + *ϵ*_4_. When |*W*_0_| > |*a*_*s*_(**n**)|, anisotropy term does not dominate the branching structure growth. When value of *W*_0_ gets smaller, it facilitates the branching structure growth, as shown in Fig 1. To study the growth of dendritic structures and nanoposts, these structures must have enough branches to have mutual interaction with nanoposts. Therefore, *W*_0_ = 0.25 is chosen in this simulation model. Due to the temperature difference of the solid region and surrounding environment, this non-equilibirum situation is considered as the driving force which gives rise to dendritic structure self-organization. When the temperature achieves the balanced temperature, the dendritic self-organized structure growth will stop (see Fig 2). At t = 60, the released latent heat accompanies the whole domain, so the solidification stops due to lack of driving force.

The branching direction of hierarchal structure is assigned as that in Karma’s phase field model [23]. In the process of structure formation, the preferred self-formation direction of hierarchal structure is 90°. From Fig 1(a)–1(c), it can be clearly seen that the main branch of hierarchal structures continuously grows mainly in 90° direction for *W*_0_ = 0.5, 0.75, and 1. While the interface thickness *W*_0_ is set to 0.25, the branch directions found are mainly 90° and 45°.

### Arrangement of nanopost arrays

**Fig 3:**
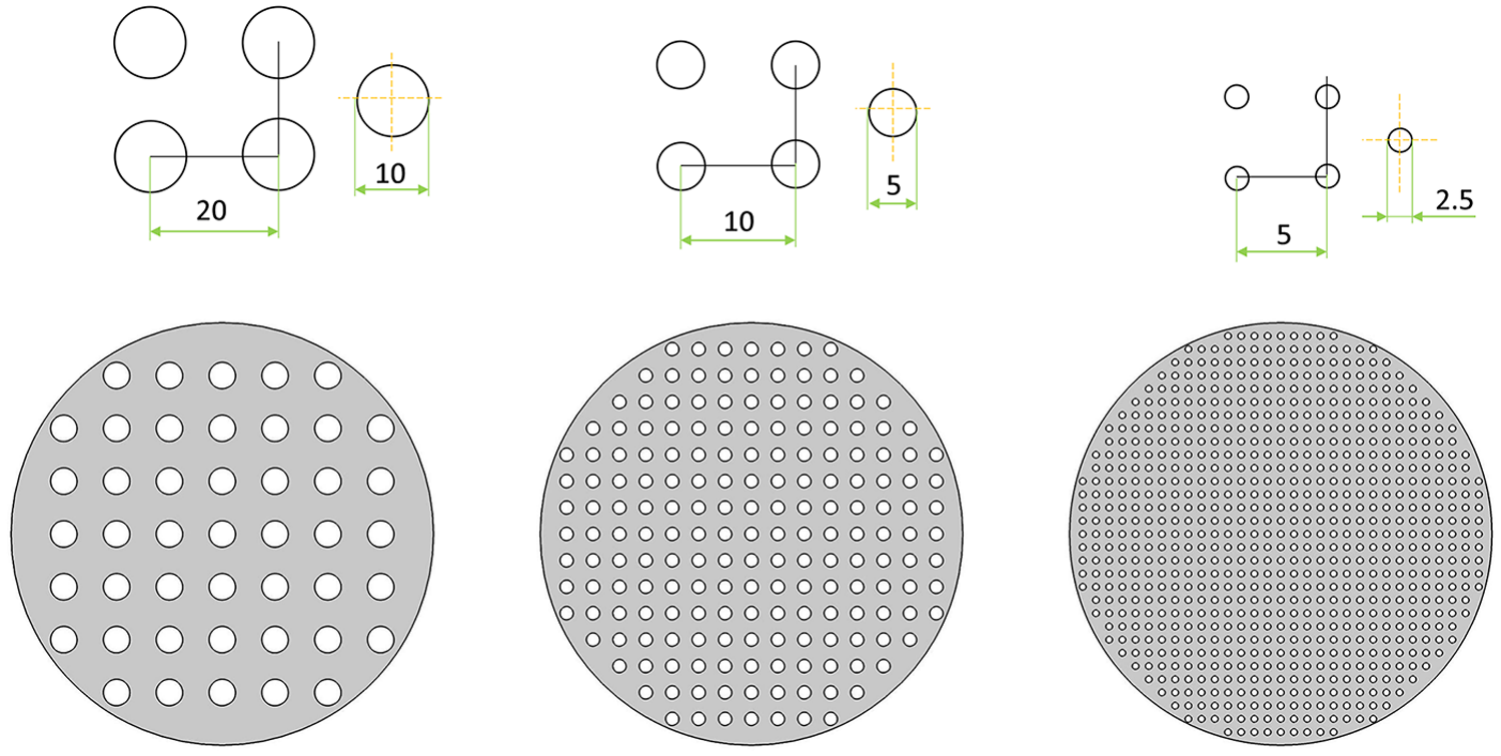
Schematics of nanopost arrays (a) Low-density of circle array, the radius of circle is 10 units and the spacing is 20 units (b) Medium-density of circle array, the radius of circle is 5 units and the spacing is 10 units (c) High-density of circle array, the radius of circle is 2.5 units and the spacing is 5 units.

**Fig 4:**
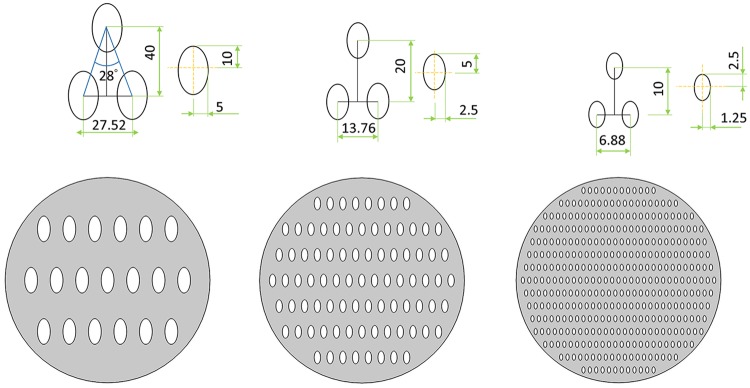
Schematics of nanopost arrays (a) Analogy for exposure angle 28 low-density elliptical arrays, the major radius of elliptical is 10 units and minor radius is 5 units, the longitudinal spacing is 40 units and lateral spacing is 19.92 units. (b) Medium-density elliptical arrays, the major radius of elliptical is 5 units and minor radius is 1.5 units, the longitudinal spacing is 20 units and lateral spacing is 9.96 units. (c) High-density elliptical arrays, the major radius of elliptical is 2.5 units and minor radius is 1.25 units, the longitudinal spacing is 10 units and lateral spacing is 4.98 units.

**Fig 5:**
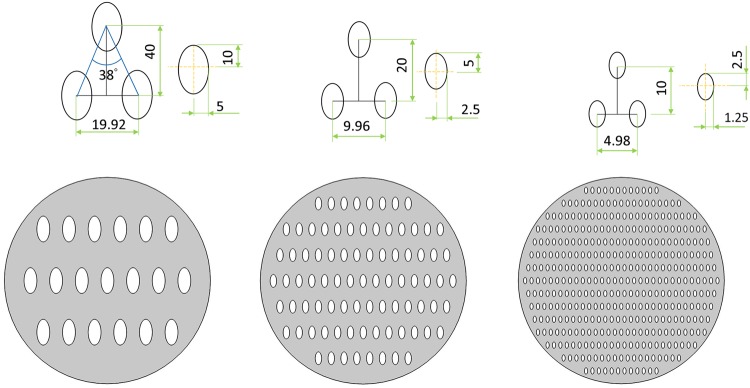
Schematics of nanopost arrays (a) Analogy for exposure angle 38 low-density elliptical arrays, the major radius of elliptical is 10 units and minor radius is 5 units, the longitudinal spacing is 40 units and lateral spacing is 27.52 units. (b) Medium-density elliptical arrays, the major radius of elliptical is 5 units and minor radius is 1.5 units, the longitudinal spacing is 20 units and lateral spacing is 13.76 units. (c) High-density elliptical arrays, the major radius of elliptical is 2.5 units and minor radius is 1.25 units, the longitudinal spacing is 10 units and lateral spacing is 6.88 units.

To study how nanoposts become obstacles in space and how nanoposts affect the hierarchal architecture formation, those geometrical structures are built-in to become space obstacles. We are interested in how these obstacles interfere the growth speed of dendritic self-organized structures. Based on the available structures LIL exposure fabrication can be created, two different geometric shapes are chosen as space obstacles [32]. In recent research, nanoposts created by two subsequent periodic exposure of the same UV light can be circular (if two lines rotated by an angle, *θ* = 90°), and elliptical (if two lines rotated by a non 90° angle). The shape of the nanopost is determined by the fabrication and technology costs, and structures with sharp angles are more difficult to fabricate. As shown in Figs 3–5, the shapes of circle and ellipse are chosen as the obstacles in this study [32].

Dendritic architecture can only grow in the free space inside the simulation domain. The areas of circular and elliptical shapes are forbidden for dendritic structure growth as a space obstacle in analogy to arrays of nanoposts on the substrate. In the geometric setting, the numbers and the sizes of circular and elliptical nanoposts and the spacing in between under three different densities are listed in Table 2 and Figs 3–5. The ratio of major axis and minor axis is fixed at 2 to adjust the spacing for analog LIL double exposure angle of 28 degree and 38 degree nanoposts arrays. Table 3 lists the ratio of *W*_0_ and lateral/longitudinal spacing between nanoposts at *W*_0_ = 0.25 for three different nanoposts under different densities (low, medium, and high).

**Table 2:** The total numbers of circular and elliptical nanoposts employed in the simulation domain in different densities.

	Number of Nanoposts		
Density	Low	Medium	High
ellipse28	19	85	369
ellipse38	13	61	273
circle	45	185	761

**Table 3:** The ratio of *W*_0_ and nanoposts spacing at *W*_0_ = 0.25 for three different nanoposts.

Circle	Density		
*W*_0_/spacing ratio	Low	Medium	High
Lateral spacing	0.0125	0.025	0.05
Longitudinal spacing	0.0125	0.025	0.05
Ellipse28	Density		
*W*_0_/spacing ratio	Low	Medium	High
Lateral spacing	0.0126	0.0251	0.0502
Longitudinal spacing	0.00625	0.0125	0.025
Ellipse38	Density		
*W*_0_/spacing ratio	Low	Medium	High
Lateral spacing	0.0091	0.0182	0.0363
Longitudinal spacing	0.00625	0.0125	0.025

### Effect of nanopost arrays

**Fig 6:**
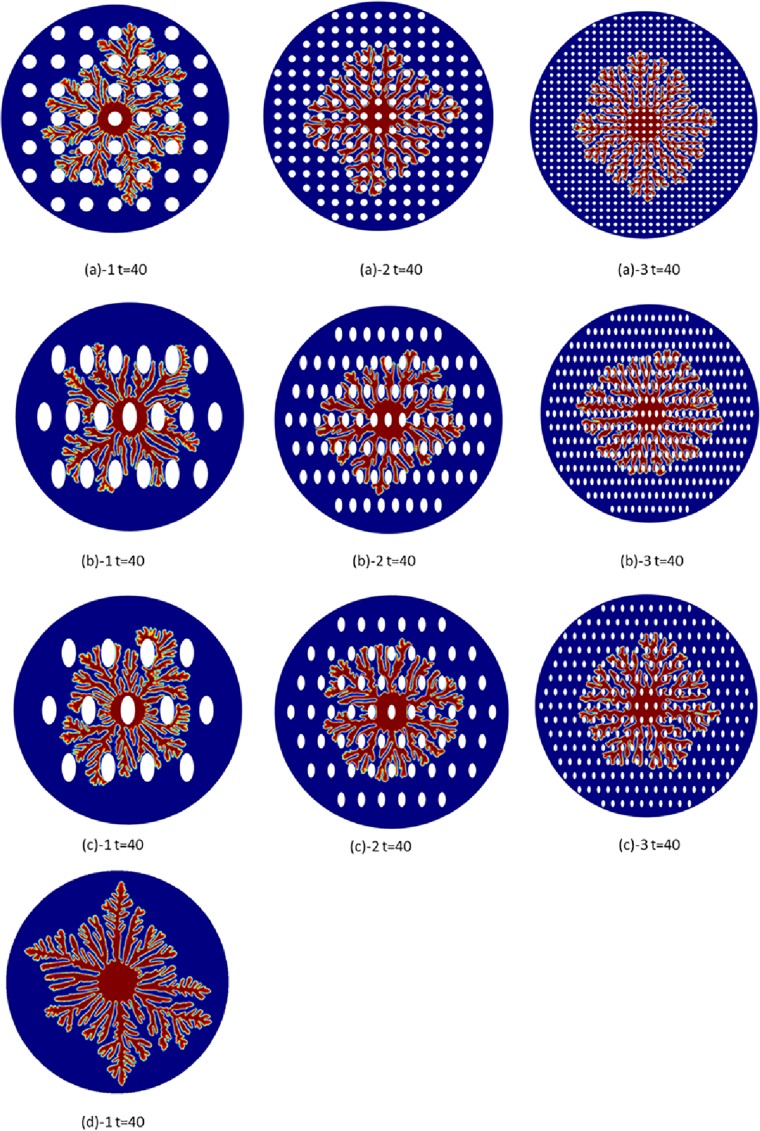
The dendritic architecture growth at t = 40 (a) circle with three different density: (a)-1: low, (a)-2:medium, (a)-3:high (b) ellipse 28 with three different density: (b)-1: low, (b)-2:medium, (b)-3:high and (c) ellipse 38 with three different density: (c)-1: low, (c)-2:medium, (c)-3:high. It is found that with increasing nanopost density the expanding velocity of the dendritic architecture is getting slower.

However, when the hierarchal architectures grow on the surface with nanopost structures guided, they will grow along the edges of nanoposts when they meet the arranged nanoposts, as shown in Fig 6, which shows the dendritic architecture growth at t = 40 for circle, ellipse28, and ellipse38 under three different densities (low, medium, and high). Fig 6(a) shows the dendritic architecture with circular nanopost guidance. Comparing with Fig 2 at t = 40, the dendritic architecture is more limited in its growth formation because of these nanopost obstacles. At low density (Fig 6(a)-1), the preferred branching angle (90°) is first blocked by the neighboring nanoposts near the initial nucleation point which causes the major branches to shift. However, the morphology of dendritic architecture still looks similar to Fig 2. At medium density (Fig 6(a)-2), the preferred branching angle (90°) is more obvious. At high density (Fig 6(a)-3), the spacing between the nanoposts is almost at the same size of the branch width. It strongly manipulates the growth path of the dendritic architecture in a very neat order. However, the expanding speed, which is the overall architecture branch tip velocity, is slow. For the circular nanopost situation, the expanding velocities of low, medium, and high density nanoposts are around 1.875, 1.75, and 1.55, respectively.

Fig 6(b) shows the dendritic architecture with ellipse28 nanopost guided. At low density (Fig 6(b)-1), the preferred branching angle (90°) is observed. However, the horizontal branch is hardly seen. With increasing nanopost density (Fig 6(b)-2 and 6(b)-3), the horizontal expanding branch is found. For the ellipse38 nanopost case, the vertical branch is more often seen since it allows more free space to grow in the vertical direction compared with the ellipse28 case. Also, with increasing nanopost density (Fig 6(c)-2 and 6(c)-3) the architecture expanding velocity decreases.

**Fig 7:**
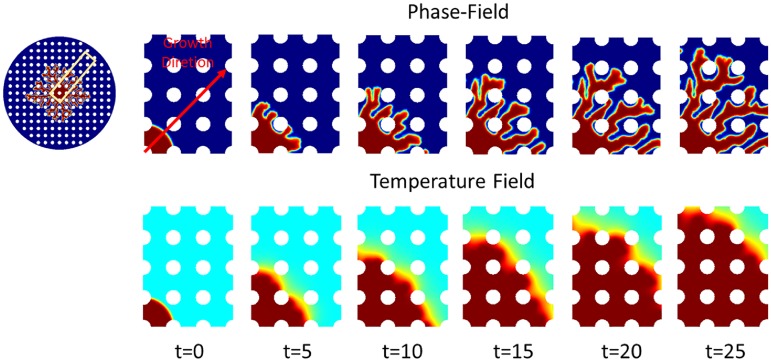
Phase field and corresponding temperature field of the dendritic structure at interface thickness = 0.25 on the surface without nanoposts. At t = 0, the center region of the simulation domain is assigned to be solid state (*ψ* = 1). Due to the temperature difference of solid region and surrounding environment, there is a driving force which gives rise to the dendritic architecture self-organization. As long as the temperature achieves the balanced temperature, the dendritic self-organization growth will be stopped.

When interface along the sides of nanoposts finds free space in a preferred direction, it will keep growing in a architecture-preferred direction. It means the formation path on the surface with nanopost structures is longer than the surface without structures. The hierarchal architecture expanding speed is getting slower because of these space obstacles. It is found that with increasing nanopost density the expanding velocity of the dendritic architecture decreases. With increasing nanopost density on the surface and decreasing spacing between nanoposts, the expanding velocity of hierarchal structure on the surface decreases. In Fig 7, the formation starts from the center of simulation domain and extends its growth outwards to the boundary. The ratio between the hierarchal structure interface thickness *W*_0_ and the spacing between nanoposts is equal to 0.025, which means the interface thickness is only 5% of the nanopost spacing. In other words, 2.5% of the hierarchal structure in nanopost spacing as a unit affects the branching ability of total structures. There are 7 circular structures in the length of radius which means the maximum numbers for hierarchal structure to contact the nanopost is 7, and the diameter of circular nanopost is 5 units. In this simulation, the initial circular radius is 10 units, and the radius of the total simulation domain is 80 units. The hierarchical architecture needs to cross 6.5 circular nanoposts if it grows along the direction of 90 degrees. There is about 63.8% of path that cannot go in a straight direction and needs to bypass the edges of circular nanoposts. The length of path is at least larger than half of the circumference (1.37 times). From Fig 7, it is seen the structure’s expanding speed is faster in the horizontal direction than the vertical direction. There are fewer dendritic structures growing in the vertical direction, which makes the whole structure distribution shift in the vertical direction.

Other than nanopost structures with different shapes, the dislocation arrangement is formed from two neighboring arrays of elliptical nanopost structures. The lateral and longitudinal spacings between nanoposts are different. It is observed that the horizontal hierarchal structures on the surface with elliptical nanoposts look similar to structures on the surface with circular nanoposts. However, the branching in vertical direction is shifted because of the array arrangement of nanopost structures. When the shape of nanostructure changes from circular to elliptical, the major axis is parallel to the vertical direction. It means that it takes a longer time to form structures in the vertical direction with respect to the spacing in between two nanoposts. Take nanopost ellipse28 surface for example, when dendritic structures meet elliptical nanoposts, these elliptical nanoposts become spatial obstacles in the 45° direction. These expanding dendritic structures can only choose either horizontal or vertical direction until the next bigger spacing to look for the possibility of structure formation in the 45° direction.

**Fig 8:**
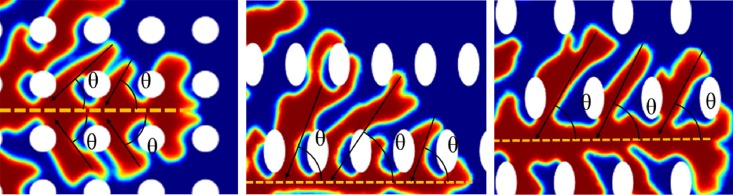
Branch angles of dendritic self-organization architecture on nanosubstrate.

**Fig 9:**
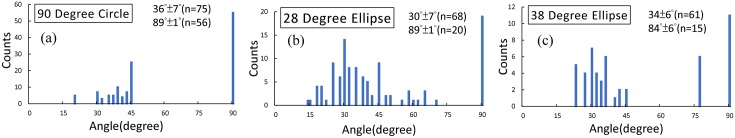
Distribution of branch angles of dendritic architectures with nanopost guided. That is, the angle between the primary-branch and the secondary-branch of the dendritic architecture. Histograms of branch angles on (a) 90°, (b) 28°, and (c) 38° nanopost substrates. The distributions show twin peaks. The first peak is around 90°, and the second peak is 36° ± 7° for 90 degree circle, 30° ± 7° for ellipse28, and 34° ± 6° for ellipse38.

In addition, it is also revealed that the branching of hierarchal structures is dependent on the array arrangement of nanoposts. Comparing the morphology of hierarchal structures on the surface with circular nanoposts and elliptical nanoposts, it is found the branching distribution is different between the two. To quantify the branching distribution, the angle between the primary-branch and secondary-branch of dendritic architectures is determined and analyzed [2]. Fig 8 shows how the branch angles are measured. In Fig 9, the angle distribution of dendritic architecture and nanopost appeared bimodal at all three nanoposts array simulations. The first peak is close to 90°, which corresponds to an assigned preferred direction. In Fig 9(a), the majority of branching angles are mainly 90°, which indicates that branches are orthogonal to each other. However, the majority of branching angles for ellipse28 and ellipse38 fall near 30° and 34° in Fig 9(b) and 9(c), respectively. The second peak (36° ±7° for 90 degree circle, 30° ±7° for ellipse28, and 34° ±7° for ellipse38) exhibits accumulated encounters between the dendritic self-organized architecture and the nanopost structure along the branch.

### Two initials on the surface

**Fig 10:**
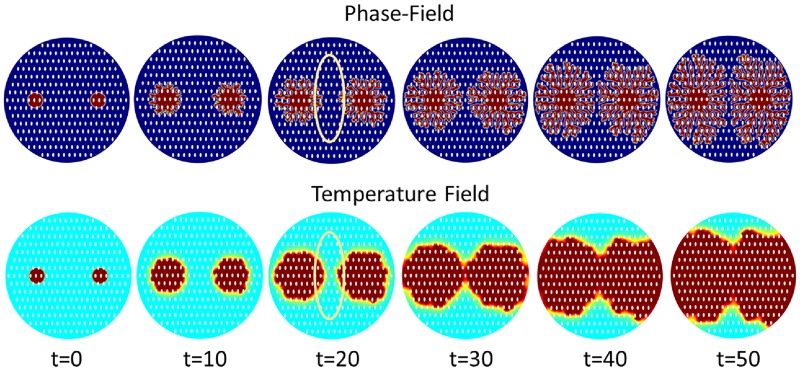
Two initial nucleation points on the surface: These two structures will try to be close to each other. Because the thermal diffusion rate is faster than the dendritic structure growing speed, the surface will achieve the thermal equilibrium very quickly which causes a distinct boundary between two structures since growing structures needs a temperature-gradient driving force.

**Fig 11:**
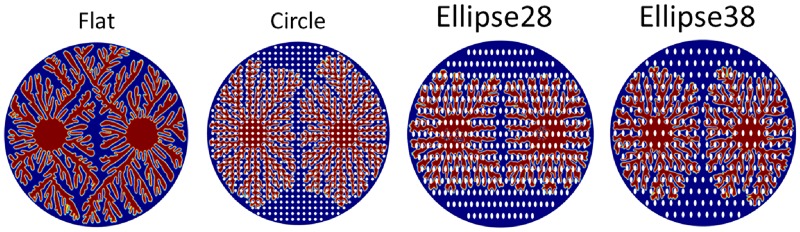
Dendritic structures with two initial starting points at the surface without nanopost and with three different kinds nanopost at t = 60. It is found these two dendritic architectures are discontinuous on the surface without nanoposts, however, there will be a distinct boundary shown between two structures on the surface with nanoposts.

With the fabrication of nanoposts, a large-area nanopost structure can be formed. When the dendritic architectures start to form, it can be seen that multiple initial points form at the same time [2]. To study the multiple initials situation, two initial points are imposed to form the dendritic structures using the same simulation conditions. From Fig 10, it can be seen that when there are two initial nucleation points on the surface, there will be two discontinuous dendritic architectures. When the boundaries of two expanding structures start to near each other, the speed of heat balance is faster than the speed of structure expanding, which leads to two initial points forming discontinuous structures. With the same conditions, two nucleation seeds are initialized on the surface with different nanopost array structures. Because nanopost structures lead to the hysteresis of dendritic structure expanding, a distinct boundary between two dendritic structures can be found also in a different nanopost array surface. Fig 11 shows the dendritic structures with two initial points at the surface without nanoposts and with three different kinds of nanoposts at t = 60. It is found that two dendritic architectures are discontinuous on the surface without nanoposts. However, there is a distinct boundary shown between two structures on the surface with nanoposts.

### Effect of active nanopost heating source/sink

**Fig 12:**
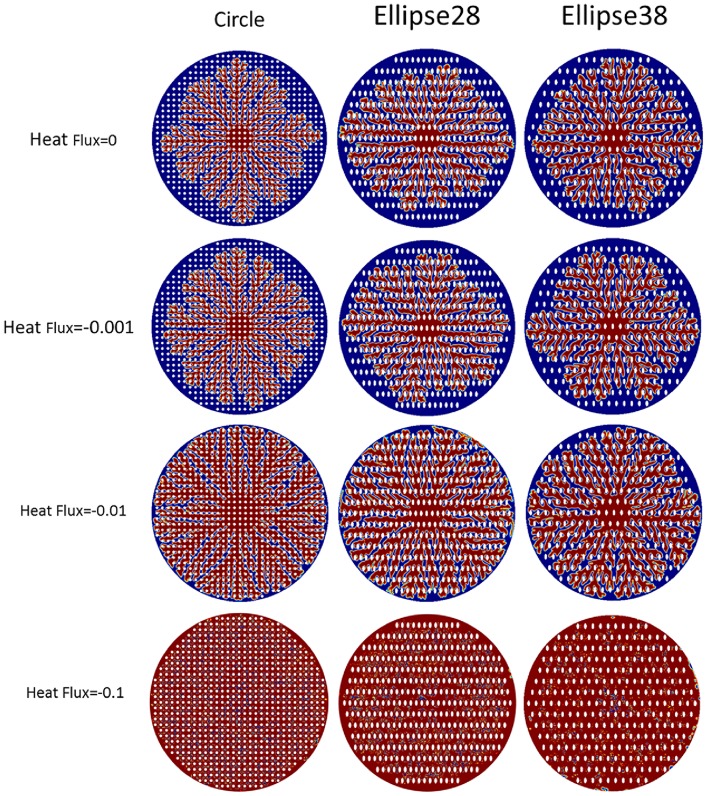
Phase contours in three different nanopost arrays when negative heat flux is introduced to the nanopost at t = 60. As long as the heat flux is getting large, solid because of the external driving force will try to fill up the gaps. It is shown solid phase distribution on the surface can be adjusted with external heat flux introduced.

In this phase field model, the temperature difference between the interface of dendritic structures and the surrounding environment is the driving force which initiates the dendritic architectures to form. When a negative heat flux is led into the system, it keeps cooling the surface to assure the driving force forming dendritic structure on the surface. On the other hand, when a positive heat flux is introduced into the system, the surface temperature approaches the equilibrium temperature. Because of the material anisotropic property, the self-organized dendritic structure cannot fill all the gaps. There will be lots of gaps that cannot be filled while the dendritic structures grow on the surface with nanopost arrays. When the heat flux is introduced into the boundaries of nanopost arrays, it allows nanostructures to change the surface temperature fields. It can be seen that the gap will be filled by dendritic structures thoroughly as the heat flux is introduced to the system. This demonstrates that an external energy excitation is needed to get the structure filled up in the whole space when material is anisotropic (see Fig 12).

**Fig 13:**
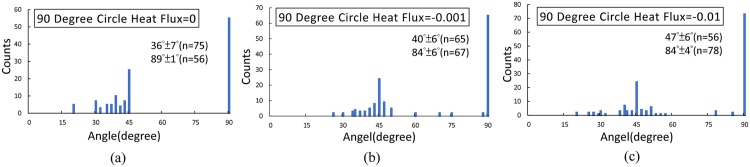
Distribution of dendritic architecture branch angles, that is, the angle between the primary-branch and the secondary-branch. Histograms of dendritic architecture branching angles on 90 degree circle with (a) no heat flux (b) heat flux = -0.001 and (c) heat flux = -0.01. The distributions still show identical twin peaks. The first peak is around 90°, and the second peak is 36° ± 7° for no heat flux, 40° ± 6° for heat flux = -0,001, and 47° ± 6° for heat flux = -0.01.

**Fig 14:**
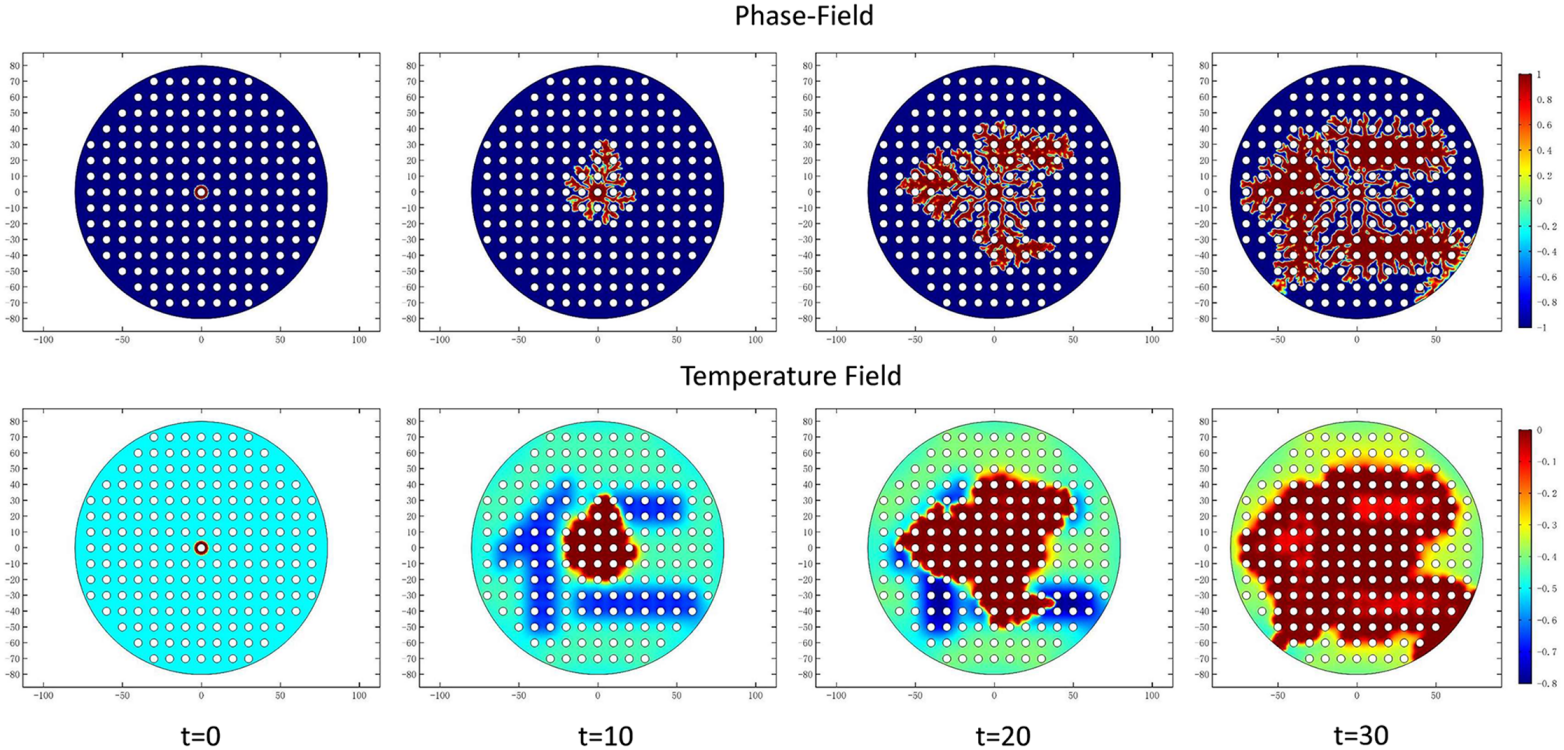
When a negative heat flux introduced at the specific nanopost structure, it can induce the Chinese character (pronunciation: Ren) formed at the area with lower temperature. When the dendritic structure extends to the lower-temperature area, solid phase intend to grow in a lower-temperature field and fill up the whole low-temperature area and then form Chinese character comparing to the area without any negative heat flux introduced which can provide an indicator for the development of smart self-organized architecture.

To quantify the effect of induced heat excitation to dendritic architecture branching distribution, the branch angles are also determined and analyzed shown in Fig 13. The branching distributions still show identical twin peaks. The first peak is around 90° which is the preferred direction, and the second peak is 36° ± 7° for no heat flux, 40° ± 6° for heat flux = -0.001, and 47° ± 6° for heat flux = -0.01. While the magnitude of negative heat flux increases, it reveals that the dendritic self-organized architectures preferred direction which is 90° dominates and the ability of nanopost array arrangement deceases. The dendritic structure will be restrained and lose the ability to become solid because of positive heat flux is induced. In this model, the change of the temperature field decided by the nanoposts with introducing heat excitation can further induce the distribution of the solid phase. When the heat flux is imposed at the specific nanopost structures, it can induce the desired Chinese character arrangement after the interaction between the dendritic structures and nanopost structures (see Fig 14). The negative heat fluxes = -0.1 are imposed at the nanoposts where the strokes of the Chinese character are assigned, and the positive heat fluxes = 0.03 are imposed at the rest of the nanoposts. It can be seen that the negative flux helps the liquid become solid, which causes the desired pattern, while the positive heat flux helps the liquid remains at the liquid state.

## Conclusion

From the simulation results, employing nanopost as a passive component induces hierarchal architecture to grow not only through the designed distribution to induce dendritic self-organization, but also further become an active component through an external driving force to affect the self-organized architecture distribution when a specific mutual interaction is applied to the nanopost surface. This extended phase field model qualitively supports the experimental results, and facilitates the self-organized architecture growth [2]. It is also found that the formation of dendritic structures is mainly based on its isotropic property, and the geometric constraint generated by nanopost arrays is another important key to induce architecture morphology change. The distribution of branch angles can be predicted by nanopost array arrangement [2] which shows two identical peaks in both experiment and simulation. In addition, when nanoposts are imposed with external driving excitations, an interaction with thermal field enhances self-organized architecture in guided pattern. The development of new and even more complex models including more dynamics can be continued based on this model. This extended phase field model developed using adaptive finite element method can be employed to fundamentally study the dynamic physics of dendritic self-organized architectures under controls of certain excitation and tailored guided configuration. It may serve as a framework for the development of smart self-organized architecture design for a large scale fabrication at low cost and energy consuming.

## References

[1] Reyes D. R., Folch A., Minhas H. & Gaitan M. The Art in Science of MicroTAS: the 2013 edition. Lab on a Chip. 2014;14(8):1389–1390. doi: 10.1039/c4lc90017k 24615301 10.1039/c4lc90017k

[2] Chang E. C., Hsu Y. R., Fu C. C., Wang Y. L., Cheng C. M. & Chen C. C. Nanopost-Guided Self-Organization of Dendritic Inorganic Salt Structures. Langmuir. 2014;30(36):10940–10949. doi: 10.1021/la502939g 25149642 10.1021/la502939g

[3] Zuppiroli L., Si-Ahmed L., Kamaras K., Nuesch F., Bussac M. N., Ades D., et al Self-assembled monolayers as interfaces for organic opto-electronic devices. Eur. Phys. J. B. 1999;11(3):505–512. doi: 10.1007/s100510050962

[4] Parviz B. A., Ryan D. & Whitesides G. M. Using self-assembly for the fabrication of nano-scale electronic and photonic devices. IEEE Trans. Adv. Packag. 2003;26(3):233–241. doi: 10.1109/TADVP.2003.817971

[5] Morris C. J., Stauth S. A. & Parviz B. A. Self-assembly for microscale and nanoscale packaging: Steps toward self-packaging. IEEE Trans. Adv. Packag. 2005;28(4):600–611. doi: 10.1109/TADVP.2005.858454

[6] Garcia-Ruiz J. M., Melero-García E. & Hyde S. T. Morphogenesis of Self-Assembled Nanocrystalline Materials of Barium Carbonate and Silica. Science. 2009;323(5912):362–365. doi: 10.1126/science.1165349 19150841 10.1126/science.1165349

[7] Garcia-Ruiz J. M., Hyde S. T., Carnerup A. M., Christy A. G., Van Kranendonk M. J. et al Self-Assembled Silica-Carbonate Structures and Detection of Ancient Microfossils. Science. 2003;302(5648):1194–1197. doi: 10.1126/science.1090163 14615534 10.1126/science.1090163

[8] Dang X. N., Yi H. J., Ham M. H., Qi J. F., Yun D. S., Ladewski R. et al Virus-templated self-assembled single-walled carbon nanotubes for highly efficient electron collection in photovoltaic devices. Nat. Nanotechnol. 2011;6(6):377–384. doi: 10.1038/nnano.2011.50 21516089 10.1038/nnano.2011.50

[9] Ma Y. R., Qi L. M., Ma J. M. & Cheng H. M., Hierarchical, star-shaped PbS crystals formed by a simple solution route. Cryst. Growth Des. 2004; 4(2):351–354. doi: 10.1021/cg034174e

[10] Kurland N. E., Kundu J., Pal S. Kundu S. C. & Yadavalli V. K. Self-assembly mechanisms of silk protein nanostructures on two-dimensional surfaces. Soft Matter. 2012; 8(18):4952–4959. doi: 10.1039/c2sm25313e

[11] Imai H., Terada T. & Yamabi S. Self-organized formation of a hierarchical self-similar structure with calcium carbonate. Chem. Commun. 2003;4:484–485. doi: 10.1039/b211240j 10.1039/b211240j 12638961

[12] Imai H., Terada T., Miura T. & Yamabi S. Self-organized formation of porous aragonite with silicate. J. Cryst. Growth. 2002;244(2):200–205. doi: 10.1016/S0022-0248(02)01616-0

[13] Imai H. Self-Organized Formation of Hierarchical Structures In Biomineralization I, Naka K., Ed. Springer Berlin Heidelberg 2007;270:43–72.

[14] Chen X. Y., Wang X., Wang Z. H., Yang X. G. & Qian Y. T. Hierarchical growth and shape evolution of HgS dendrites. Cryst. Growth Des. 2005;5(1):347–350. doi: 10.1021/cg0498599

[15] Parfenov A., Gryczynski I., Malicka J., Geddes C. D. & Lakowicz J. R. Enhanced Fluorescence from Fluorophores on Fractal Silver Surfaces. The Journal of Physical Chemistry B. 2003;107(34):8829–8833. doi: 10.1021/jp022660r 20686644 10.1021/jp022660r PMC2913721

[16] Piana S., Lindorff-Larsen K. & Shaw D.E. Atomic-level description of ubiquitin folding. Proc. Nat. Acad. Sci. 2013;110:5915 doi: 10.1073/pnas.1218321110 23503848 10.1073/pnas.1218321110 PMC3625349

[17] Mishin Y., Asta M. & Li J., Atomistic modeling of interfaces and their impact on microstructure and properties. Acta Mater. 2010;58:1117 doi: 10.1016/j.actamat.2009.10.049

[18] Alster E., Elder K.R., Hoyt J.J & Voorhees P.W. Phase-field-crystal model for ordered crystals. Physical Review E. 2017;95:022105 doi: 10.1103/PhysRevE.95.022105 28297840 10.1103/PhysRevE.95.022105

[19] Mickiewicz R.A., Yang Joel K. W., Hannon Adam F., Jung Yeon-Sik, Alexander-Katz Alfredo, Berggren Karl K., & Ross Caroline A. Enhancing the Potential of Block Copolymer Lithography with Polymer Self-Consistent Field Theory Simulations. Macromolecules. 2010;43:8290–8295. doi: 10.1021/ma101360f

[20] Zhang L., Wang L. & Lin J. Harnessing Anisotropic Nanoposts to Enhance Long Range Orientation Order of Directed Self Assembly Nanostructures via Large Cell Simulations. Macro Lett. 2014;3:712–716. doi: 10.1021/mz5003257 10.1021/mz5003257 35590714

[21] Xie N., Li W., Qiua F. & Shi A.C. New strategy of nanolithography via controlled block copolymer self-assembly. Soft Matter. 2013;9: 536–542. doi: 10.1039/C2SM26833G

[22] Kobayashi R. Modeling and numerical simulations of dendritic crystal growth. Physica D: Nonlinear Phenomena. 1993;63(3):410–423. doi: 10.1016/0167-2789(93)90120-P

[23] Karma A. & Rappel W. J., Quantitative phase-field modeling of dendritic growth in two and three dimensions. Phys. Rev. E. 1998; 57(4):4323–4349. doi: 10.1103/PhysRevE.57.4323

[24] Demange G., Zapolsky H., Patte R. & Brunel M. A phase field model for snow crystal growth in three dimensions. npj Computational Materials. 2017;3:15 doi: 10.1038/s41524-017-0015-1

[25] Kozlov M. M., Kuzmin P. I. & Popov S. V. Formation of cell protrusions by an electric field: a thermodynamic analysis. European Biophysics Journal. 1992;21(1):35–45. doi: 10.1007/BF00195442 1516559 10.1007/BF00195442

[26] Mohsen A. Z. & Felicelli S.D. Comparison of Cellular Automaton and Phase Field Models to Simulate Dendrite Growth in Hexagonal Crystals. Journal of Material Science Technology.2012;28:137–146. doi: 10.1016/S1005-0302(12)60034-6

[27] Hsu H.Y, Lin B.T & Hsu Y. R. Three-dimensional numerical investigation of dendritic self-organizational structure growth on a nanopost surface. Advances in Mechanical Engineering. 2017;9(2):1–9. doi: 10.1177/1687814016683357

[28] Wang M., Liu X.Y., Strom C.S., Bennema P., Enckervort W., & Ming N.B. Fractal aggregation at low driving force with strong anisotropy. Phys. Rev. Lett. 1998; 80(14):3089–3092. doi: 10.1103/PhysRevLett.80.3089

[29] Oaki Y. & Imai H. Experimental demonstration for the morphological evolution of crystal grown in gel media. Crystal Growth Des. 2003;3(5):711–716. doi: 10.1021/cg034053e

[30] Ming N. B., Wang M. & Peng R. W. Nucleation-limited aggregation in fractal growth. Phys. Rev. E. 1993;48(1):621–624. doi: 10.1103/PhysRevE.48.621 10.1103/physreve.48.621 9960630

[31] Mu W., & Ming N. B. Insitu observation of surface-tension induced oscillation of aqueous-solution films in needle-like crystal growth. Phys. Rev. A. 1991;44(12):R7898–R7901. doi: 10.1103/PhysRevA.44.R7898 9906025 10.1103/physreva.44.r7898

[32] Chang E. C., Mikolas D., Lin P. T., Schenk T., Wu C. L., Sung C. K. & Fu C. C., Improving feature size uniformity from interference lithography systems with non-uniform intensity profiles. Nanotechnology. 2013;24(45):455301 doi: 10.1088/0957-4484/24/45/455301 24141145 10.1088/0957-4484/24/45/455301

